# Growth impairment after TBI of leukemia survivors children: a model- based investigation

**DOI:** 10.1186/1742-4682-11-44

**Published:** 2014-10-13

**Authors:** Chiara Galletto, Antonio Gliozzi, Daniele Nucera, Nicoletta Bertorello, Eleonora Biasin, Andrea Corrias, Patrizia Chiabotto, Franca Fagioli, Caterina Guiot

**Affiliations:** Pediatric Onco-Hematology, Stem Cell Transplantation and Cellular Therapy Division, Regina Margherita Children’s Hospital, piazza Polonia 94, 10126 Turin, Italy; Department of Physics, Politechnics of Turin, Turin, Italy; Department of Animal Pathology, University of Turin, Turin, Italy; Department of Neuroscience, University of Turin, Turin, Italy

**Keywords:** TBI, HSCT, GHD, Growth, Cancer survivors, Mathematical model

## Abstract

**Background:**

Children receiving Total Body Irradiation (TBI) in preparation for Hematopoietic Stem Cell Transplantation (HSCT) are at risk for Growth Hormone Deficiency (GHD), which sometimes severely compromises their Final Height (FH). To better represent the impact of such therapies on growth we apply a mathematical model, which accounts both for the gompertzian-like growth trend and the hormone-related ‘spurts’, and evaluate how the parameter values estimated on the children undergoing TBI differ from those of the matched normal population.

**Methods:**

25 patients long-term childhood lymphoblastic and myeloid acute leukaemia survivors followed at Pediatric Onco-Hematology, Stem Cell Transplantation and Cellular Therapy Division, Regina Margherita Children’s Hospital (Turin, Italy) were retrospectively analysed for assessing the influence of TBI on their longitudinal growth and for validating a new method to estimate the GH therapy effects. Six were treated with GH therapy after a GHD diagnosis.

**Results:**

We show that when TBI was performed before puberty overall growth and pubertal duration were significantly impaired, but such growth limitations were completely reverted in the small sample (6 over 25) of children who underwent GH replacement therapies.

**Conclusion:**

Since in principle the model could account for any additional growth ‘spurt’ induced by therapy, it may become a useful ‘simulation’ tool for paediatricians for comparing the predicted therapy effectiveness depending on its timing and dosage.

**Electronic supplementary material:**

The online version of this article (doi:10.1186/1742-4682-11-44) contains supplementary material, which is available to authorized users.

## Background

Growth is a finely regulated phenomenon that results from interaction of genetics, nutrition, hormones, metabolism and cerebrocortical influences: in childhood it is largely influenced by growth hormone (GH) and in puberty by the synergistic action of GH and sex steroids. Puberty is the period of human development during which physical growth completes and sexual maturation occurs; this condition implies becoming first capable of sexual reproduction and is marked by the maturation of the genital organs and the development of the sexual secondary characteristics. The normal pubertal growth rate (complete enlargement degree) is approximately 1.5–2 times greater than the prepubertal growth rate. GH stimulates growth of epiphyseal cartilage and subsequent bone growth directly by action of Insulin-like growth factor I (IGF-1). When insufficient GH is produced, growth velocity and bone maturation are delayed and the divergence of the growth rate from normal increases with age unless replacement therapy is administered [1].

High-dose chemotherapy or chemo-radiotherapy followed by hematopoietic stem cell transplantation (HSCT) used to treat children with cancer has resulted in an ever-increasing number of long-term survivors. These patients are at risk for a variety of late effects due to the disease itself, treatment exposure (surgery, chemotherapy and radiotherapy) and possible underlying medical problems. Patients who received total body irradiation (TBI) in preparation for HSCT are at risk of developing a deficiency of one or more hormones produced by the hypothalamus and pituitary gland (HP region): both these treatments are known to affect growth and development. Chemotherapy, decreased nutritional intake, hematopoietic stem cell transplantation, corticosteroids and other endocrinological complications (hypothyroidism and hypogonadism) may also affect growth in childhood cancer survivors, but to a lower extent [2–4].

The incidence of GH deficiency (GHD) after TBI and HSCT varies from 20 to 85% depending upon differences in time of testing after HSCT, differences in preparative regimen received, inclusion of patients with and without cranial irradiation and use of different methods of GH testing, as described in a recent review [1].

It has been suggested that risk factors like higher CRT (cranial radiotherapy) dose, larger fraction size or larger number of fractions, increased volume of the HP region exposed, higher biological effective dose (BED), younger age at treatment and longer follow up time affect the risk of GHD secondary to radiation of the HP region [5, 6]. In addition, gender (male sex) may also influence the prevalence of GHD. GH therapy is able to gain a total height inversely related to patient age at the start of GH treatment and positively related to its duration. Treatment with GH before the child’s height has decreased to below the third percentile results in the greatest final height response to treatment. Growth before puberty is the major determinant of final height, therefore treatment with GH during the prepubertal period needs to be optimized.

Traditionally juvenile growth is evaluated by comparison with appropriate Height Growth Charts (HGC), which report proper statistical parameters (usually the 25^th^, 50^th^ and 75^th^ centiles of the heights extracted from measurements performed in large homogeneous populations) versus age (see for instance [7–9]). In order to model the growth from early childhood to maturity, alternative approaches have been proposed [10]. Gliozzi A et al. [11] have recently presented an alternative method for the fitting and modelling of human HGC and individual (longitudinal) growth curves, based on the formalism of the Phenomenological Universalities (PUN) [12], already used in the analysis of several datasets of great relevance for growth and to verify the influence of the different parameters.

We evaluated if such a model can be useful to assess the influence of TBI on longitudinal growth. In fact, given the differences between individual timing and sequence of the pubertal events, as well as the individual response to therapies, to compare the genuine patient growth data with proper ‘growth chart’ references can be misleading. On the contrary, the model uses the data of the patients for fitting the values of a few meaningful model parameters. Some of those parameters are related to the prepubertal growth, which in most cases are not influenced by TBI and other HSCT conditioning therapies, but simply reflect the individual ‘growth potential’. Other parameters strictly describe pubertal growth, and are expected to be highly sensitive to TBI. Such values will be finally compared with those pertaining to the reference growth curve, aiming at assessing the quantitative impact of TBI on growth. The goal of the present paper is to validate this alternative method in a setting of long term leukemia survivors who underwent to HSCT and to assess if it would be able to estimate the GH therapy effects and to suggest a predictive algorithm to personalize GH therapy and to simulate its effect on the final height (FH).

## Methods

### Patients and intervention

Patients described in this monocentric retrospective study were all included in a long-term oncological and endocrinological follow-up or childhood lymphoblastic and myeloid acute leukaemia survivors performed at Pediatric Onco-Hematology, Stem Cell Transplantation and Cellular Therapy Division, Regina Margherita Children’s Hospital, Turin, Italy; these children were diagnosed at Our Centre between 15/05/1988 and 29/06/2005. Although, according to the Italian regulations, for retrospective studies that did not involve additional withdrawals but only the extrapolation of data from existing medical records and where retrospective data is anonymous authorizations are not strictly due, we required and obtained the Ethic Committee approval (see Additional file 1).

Patients were eligible for this study if they met all the following inclusion criteria: (1) having undergone HSCT for childhood acute leukaemia after a myeloablative conditioning regimen, (2) having an italian origin, (3) having undergone an oncological and endocrinological at least 5-year long follow-up after HSCT with a complete growth evaluation (4) having at least one height measurement before TBI and a follow up lasting until sexual maturation. Patients who developed a second malignant tumor or who achieved final height in a short follow up period were excluded. Hormone therapy was never administered to promote puberty.

Out of 90 patients diagnosed and treated at our hospital between 1988 and 2005, twenty-seven young long term survivors met these inclusion criteria and are described here. All patients has been followed by oncologists and endocrinologists possibly until the age of 18 and they all were in remission without complications at the time of this study. All the patients have been treated at our hospital according to the AIEOP (Associazione Italiana Ematologia-Oncologia Pediatrica-Italian Association of Pediatric Onco-Hematology)-BFM (Berlin–Frankfurt–Munster) ALL and AML Protocols, and had received TBI as part of their conditioning regimen before HSCT. Patients features are summarized in Table 1. Further details about patients who underwent GHT are given as Additional file 1.

**Table 1:** **Detailed list of the patients considered in the present study**

Patient	Sex	Age at TBI (years)	GH therapy (Y/N)
1	F	8.4	N
2	F	1.1	N
3	M	10.4	N
4	M	5.0	N
5	M	6.3	N
6	F	6.9	N
7	F	7.0	N
8	M	7.3	N
9	F	13.5	N
10	M	10.6	N
11	M	6.9	Y
12	F	11.2	N
13	F	4.9	Y
14	M	14.2	N
15	F	12.7	N
16	F	6.8	Y
17	F	6.3	Y
18	M	8.0	N
20	M	4.9	Y
21	M	5.3	N
22	F	2.8	N
23	M	12.2	N
24	F	7.3	N
25	M	13.0	N
27	F	9.3	Y

Two female patients, one not (n. 19) and one (n. 26) undergoing GHT, were excluded by the samples because the estimated values were not reliable. In the first case the number of available data was too small to get a reasonable fitting of the parameters, in the second case we realized that GHT, delivered between 8 and 9 years of age, was very effective and the corresponding growth spurt was misinterpreted by the numerical program as the age of puberty.

### Preparative regimens to HSCT

The preparative regimen depended on the protocols in use at the time of transplantation, the underlying disease and its status, and the existence or not of previous central nervous system (CNS) irradiation. TBI was always administered fractionated and all patients received the same total dose of 12 Gy (that is, 2Gy twice daily during 3 days). TBI was associated to etoposide, to etoposide and cyclophosphamide, to melphalan (5 patients respectively), to thiotepa and cyclophosphamide (5 patients), to cyclophosphamide and to fludarabine (1 patient in both cases), to fludarabine and thiotepa (2 patients).

### Growth evaluation

Height was measured at first diagnosis, at the beginning of the second line therapy in case of leukemia relapse, at starting of preparative regimen to HSCT and then every six/twelve months until 18 years of age, as part of endocrinological examinations follow up. From these measurements it was possible to obtain information on the pubertal growth in terms of overall growth (complete enlargement) and growth velocity (heightening speed; cm/year). Standing height, measured using a Harpenden stadiometer, was used for all patients. Final height (FH) was defined as the tallest height measured when the patient’s age was 18 years or older, and when height velocity was less then 1 cm per year.

### Growth hormone deficiency (GHD)

The diagnosis of GHD in childhood is a multifaceted process requiring comprehensive clinical and endocrinological assessment, combined with biochemical tests of the GH-insulin-like-growth factor (IGF) axis. Criteria to initiate investigation include: 1) severe short stature, defined as a height more than 3SD below the mean; 2) height more than 1.5 SD below the midparental height; 3) height more than 2SD below the mean and a height velocity over 1 yr more than 1 SD below the mean for chronological age, or a decrease in height SD more than 0.5 over 1 yr in children over 2 yr of age; 4) in the absence of short stature, a height velocity more than 2 SD below the mean over 1 yr or more than 1.5 SD sustained over 2 yr.

Growth hormone (GH) deficiency was detected by measuring insulin-like growth factor I plasma levels and GH peak response to at least two stimulation tests per patient (first test: Arginine, second test glucagon or insulin). GH insufficiency was diagnosed when peak GH levels after stimulation were inferior to 10 mcg/L [13].

For patients who underwent a GH therapy we considered: length at birth, target height; somatomedins and other biochemical abnormalities at the start of therapy; age at beginning of GH therapy dose and duration. Nobody had thyroid disfunction; only one patient had gonadal disfunction (patient 6) (see Additional file 1).

### Physical model

Growth is described, according to [11] and the parameters listed in Table 2, using an algorithm which assumes that, starting from an initial height y_0_, (actually corresponding to 3-months stature) a gompertzian [14] lengthening with growth rate a_0_ and carrying capacity related parameter k_0_ occurs.

**Table 2:** **Detailed list of parameter values evaluated by the model**

	Case	Y _0_ (height at 3 months in cm)	k _0_ (carrying capacity)	a _0_ (pre-pubertal growth rate)	t _m_ (age at puberty)	y _1_ (overgrowth due to puberty in cm)	a _1_ (pubertal growth rate)	σ (puberty time span in yrs)
	1	62.39727	0.190254	0.218913	11.84713	12.22394	4.497026	0.591718
Females	2	56.18536	0.26937	0.276819	10.50654	8.135071	1.232418	1.748695
	6	58.33898	0.248981	0.294922	11.18104	11.66339	1.030791	1.519584
	7	63.2854	0.1975	0.2324	9.655209	7.9907	7.2763	0.3051
	9	62.229	0.192012	0.217	10.25788	9.588047	1.399609	1.212012
	12	62.33923	0.192248	0.224598	13.43577	6.946294	6.07616	1.31218
	15	63.92922	0.172435	0.198459	10.84313	5.841731	9.715973	0.577892
	22	62.3936	0.1903	0.2189	10.85878	12.2237	4.4767	0.5918
	24	53.83767	0.257778	0.274054	12.86398	9.008473	7.301078	1.27438
	**13**	**70.89**	**0.097948**	**0.110792**	**10.16677**	**5.630503**	**4.339571**	**0.667967**
	**16**	**73.39873**	**0.08876**	**0.119807**	**12.92137**	**18.89658**	**5.471942**	**0.17205**
	**17**	**61.28031**	**0.19219**	**0.271627**	**11.60383**	**28.09531**	**0.869462**	**4.42738**
	**27**	**60.32773**	**0.201376**	**0.217701**	**11.45546**	**2.54826**	**1.79574**	**0.001265**
	Reference values 50th centile HGC	61.94	0.187	0.218	10.31	15.24	0.61	2.01
	3	72.35844	0.101311	0.135207	13.7949	7.091698	1.333014	0.670117
Males	4	68.32549	0.146165	0.189528	20.01918	3.759593	4.040935	3.719143
	5	66.97872	0.146127	0.161664	12.5558	6.622826	2.100794	0.592351
	8	71.88692	0.10269	0.122161	12.89852	8.895164	4.251733	1.715174
	10	65.03691	0.164741	0.169928	11.92245	6.199538	9.831924	0.705535
	14	61.74667	0.212759	0.244471	11.54411	15.17584	0.679802	2.016547
	18	67.1999	0.129419	0.14843	13.2384	11.72742	2.069234	0.817563
	21	78.24132	0.065035	0.090613	14.88816	17.0507	1.35473	0.851504
	23	69.22207	0.137272	0.154906	15.0422	6.979829	2.339744	0.525147
	25	69.0407	0.138559	0.148518	12.96021	13.12342	-0.18501	1.492829
	**11**	**66.39639**	**0.148124**	**0.204369**	**12.50947**	**11.13404**	**1.148798**	**1.087921**
	**20**	**75.0882**	**0.099678**	**0.123619**	**11.29312**	**6.9798**	**2.3397**	**0.5251**
	Reference values 50th centile HGC	67.82	0.136	0.149	12.79	14.73	0.84	1.45

Patients reported in bold underwent TBI+GH therapy.

Following endogenous hormones production, growth shows accelerations or ‘spurts’ at mid-childhood and at adolescence. The last, concomitant with puberty, is the predominant one and can be modeled as a Gaussian-like velocity spurt with growth rate a_1_ occurring at the average age of t_m_, and with a time distribution given by a variance σ, which causes an overgrowth y_1_.

The above model can be expressed by the Eq:
1

being G the Gauss cumulative function:
2

The above equations can be used for fitting individual growth data (longitudinal studies) as well as HGC (transversal studies) [7–9]. A dedicated MATLAB^®^ program fitted the parameter values which best interpolate the growth curve of the model with the available data describing the height of each patient. A more exhaustive explanation of the mathematical procedure can be found in Gliozzi et al. [11].

### Statistical analysis

The difference Δ between the values estimated by the model for each patient given in Table 2 and the 50^th^ centile of appropriate HGC has been computed for each variable. The sample size is given in brackets. The mean and the related 95% Confidence Interval (95% C.I.) of Δ were calculated for all the patient of the study (N = 25), for those undergoing TBI but not GH therapy (N = 19) and for male (N = 10) and female (N = 9) separately. Similar statistics were calculated for the group of children who received both TBI and GH therapy (N = 6). In this analysis gender was not considered for the limited sample size available. Whenever the range of Δ corresponding to the 95% C.I. did not overlap the vertical line of null values, the result was considered significantly (p = 0.05) different from the null hypothesis. The central point of the Δ range is the mean value of the difference between the parameter value evaluated in the sample and in the reference population: being it negative (positive) means that the parameter is smaller (larger) of the given extent than expected.

## Results

**Figure 1:**
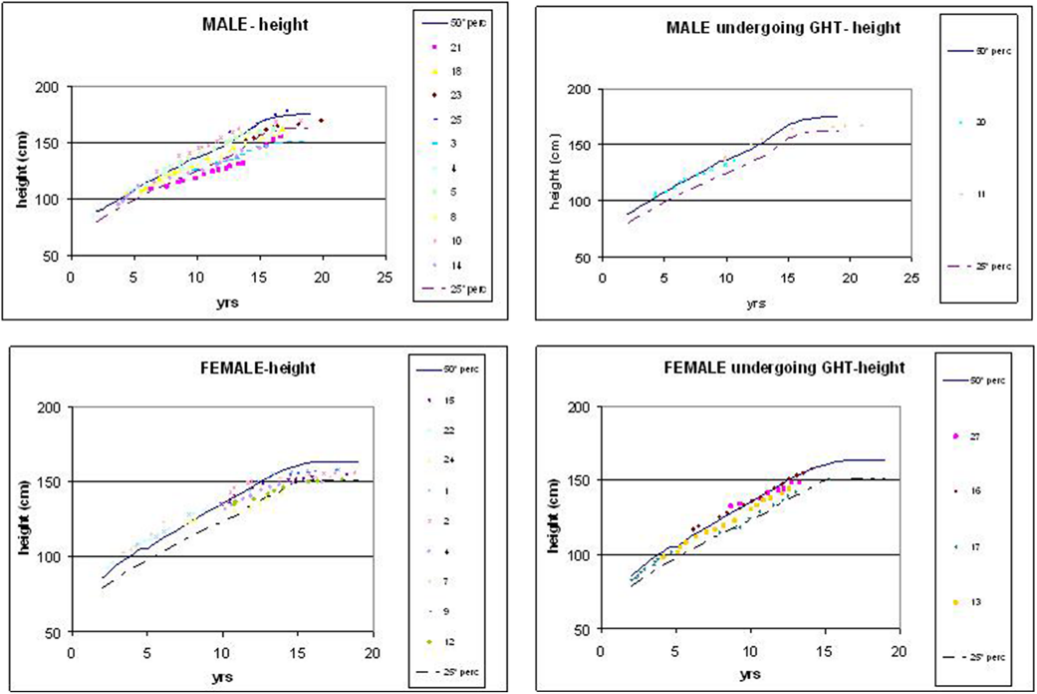
**Reports the height of the 12 males and 13 females considered in the present study, comparing their stature with the 50-th and 25-th centile of appropriate HGC. Patients are divided between those who have not (on the left) and those who have (on the right) undergone Growth Hormone Therapy (GHT).**

Figure 1 (a and b) reports the height of the 12 males and 13 females included in the present study, comparing their stature with their reference, i.e. the 50-th centile of appropriate HGC. The 25-th centile is reported as well. It shows that growth is severely impaired and the Final Height is always lower than the reference values except for one case (male n.25).

**Figure 2:**
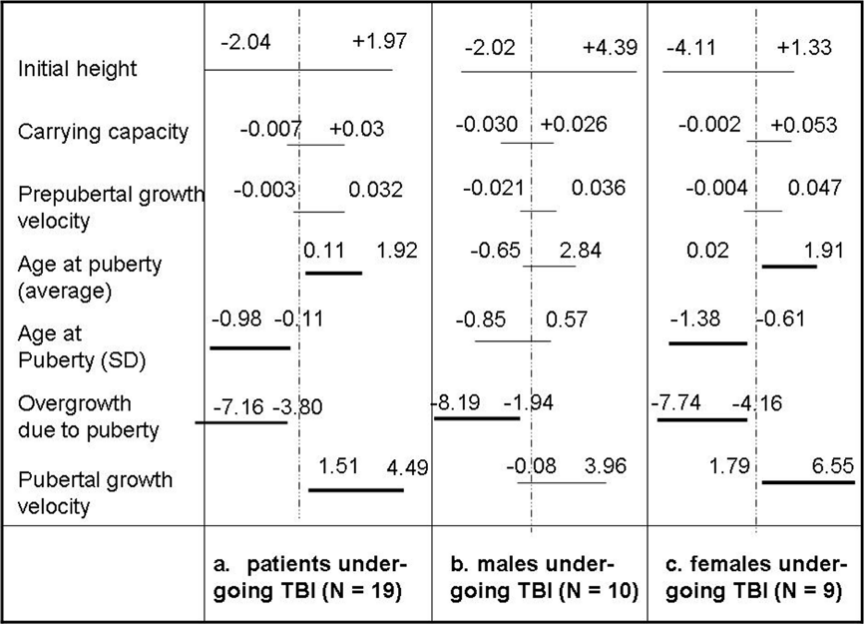
**Plot of the 95% Confidence Interval of the variable Δ = value estimated by the model on the sample - value estimated by the model on the 50** ^**th**^ **centile of appropriate HGC for all the model parameters.** The considered samples are the total of the patients undergoing TBI but not GH therapy **(a)**, the subsample formed by the males **(b)** and by the females **(c)**.

As shown in Figure 2, assuming as reference the 50^th^ centiles of appropriate HGC, we considered the patients undergoing TBI without a following GH therapy (n = 19) (b) 10 males and 9 females undergoing TBI (c) and we compared them to their reference. The statistical analysis showed that the 19 patients undergoing TBI exhibited a normal pre-pubertal growth but significantly differ from their reference in the overall pubertal growth (-5.5 cm), in the pubertal growth velocity (+3.0 cm/yr) and in the average (+1.0 yr) and standard deviation of the time of pubertal growth (-0.5 yr). Moreover, when the genders were separately considered, all the above parameters were still significantly different from normal for females, while the males who underwent TBI only show a reduced pubertal growth in comparison with their reference.

**Figure 3:**
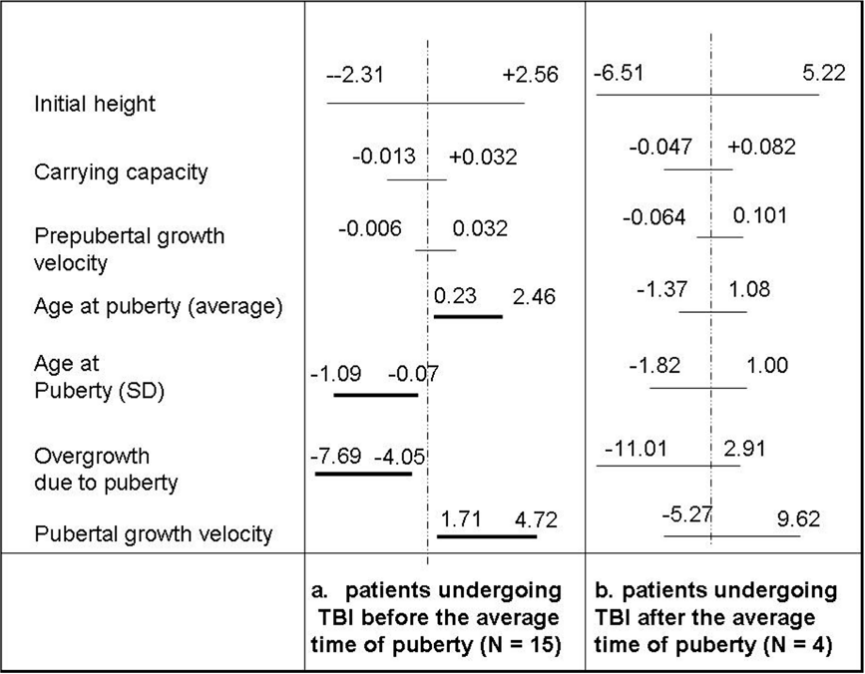
**Plot of the 95% Confidence Interval of the variable Δ = value estimated by the model on the sample - value estimated by the model on the 50** ^**th**^ **centile of appropriate HGC for all the model parameters.** The considered samples are the patients undergoing TBI before **(a)** and after **(b)** the average time of puberty.

Comparing patients undergoing TBI before (n = 14) (Figure 3a) and after the average time of puberty (n = 5) (Figure 3b) versus the respective reference, we observed that patients undergoing TBI before puberty (n = 14) showed a delayed mean pubertal age (+1.34 yr), a reduced pubertal growth (-5.9 cm), an increased post-pubertal growth velocity (+3.2 cm/yr) and a shorter value of the standard deviation of the pubertal time (-0.6 yr). Those undergoing TBI later than puberty showed a normal growth.

**Figure 4:**
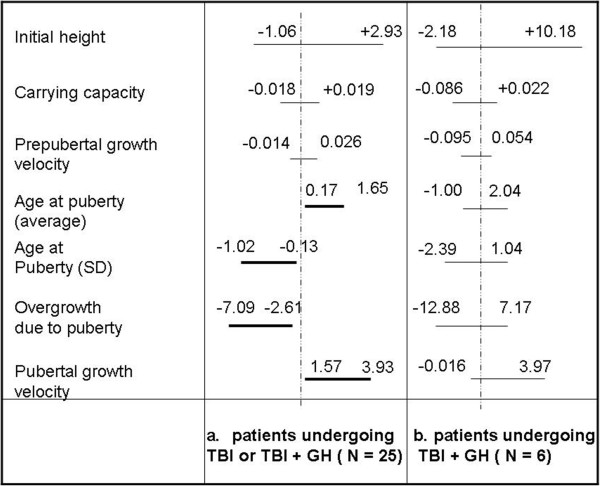
**Plot of the 95% Confidence Interval of the variable Δ = value estimated by the model on the sample - value estimated by the model on the 50** ^**th**^ **centile of appropriate HGC for all the model parameters.** The considered samples are the patients undergoing only TBI or TBI + GH therapy **(a)** and those undergoing TBI + GH therapy **(b)**.

Six patients (4 females and 2 males) received GH therapy after TBI and HSCT. As described in Figure 4, if we add to the previous sample (N = 19) also these patients, the same parameters which significantly differed from normal population still did so. Due to the reduced proportion (6 over 25) it seems conceivable that the parameter values evaluated for this sample has a low ‘weight’ in comparison with those pertaining to the other 19 patients who didn’t undergo any GH replacement therapy.

Finally, comparing growth after TBI (N = 19) with TBI + GH therapy (N = 6), the statistical analysis showed that no one of the above considered parameter significantly differed anymore from those pertaining to the normal population. GH therapy proved therefore effective in reversing the effect of TBI on pubertal growth. Such a ‘catch up’ effect is quite interesting and, although few (N = 6) cases have been studied up to now, gives a very positive feedback about the effectiveness of the GH replacement therapy administered in our clinical context.

## Discussion and conclusions

Intensive chemotherapy regimens with or without CNS irradiation are associated with persistent growth impairment [1, 2, 15–17], a multifactorial process implicating first-line treatments, post transplant complications and their consecutive treatments, prolonged use of steroid for GVHD and myeloablative conditioning regimens [18–22] either following TBI or cranial radiotherapy, which has similar effects on the hypothalamus and pituitary gland. Conditioning regimens seem to alter growth throughout the combined effects of lesions of the hypothalamic-pituitary gland axis, multiple endocrine dysfunction (thyroid and gonadal) and damage to the bone epiphyses [1, 23–27].

In our study 25 long-term leukemia survivors were retrospectively analyzed for assessing the influence of TBI on their longitudinal growth and for validating a new method to estimate the GH therapy effects. Six were treated with GH therapy after a GHD diagnosis.

The values of the parameters entering the model were estimated for all the patients and compared with the values corresponding to the 50^th^ centile of the appropriate HGC. All the values of the parameters referring to the prepubertal status (y_0_, a_0_, k_0_) were never significantly different from those of the normal population, confirming that TBI and all the related therapies were delivered to a sample extracted by the general population. On the contrary, TBI impacted on the post-puberal parameters both for males and females and also GH therapy following TBI and HSCT proved effective in compensating TBI-induced growth limitation. Our analysis confirms that TBI severely affects the post-pubertal growth parameters, mainly the overall growth and the pubertal duration, expecially when it was performed before puberty [28]. Although only a very small sample was available, we also showed that the above growth limitations are significantly reduced when GH therapy follows TBI. Furthermore, a possible bias of the program has to be assessed, since the program interprets any growth discontinuity, maybe due to effective GHT, as puberty. A careful check of the data should always been performed before processing.

In conclusion, the value of the post-pubertal parameters have been shown to be changed by GH therapy but, due to the small number of patients included in the present study, we were unable to relate those value to very important factors, such as the specific drug and dosage, the age at which the therapy was started, its duration, etc. At the moment we can just speculate that such mathematical approach can open a new working prospective towards ‘personalized treatments’. A larger, possible multicentric, study could in principle discriminate all the above factors and evaluate their specific different impact on the model parameters. At that time, being the model able to produce longitudinal curves on the basis of the values of those parameters, it could be used as a sort of “clinical simulator” in order to predict the final effect of any specific dose of GH and, even more remarkably, of the timing of the therapeutic treatment, helping pediatricians and endocrinologists to find the best clinical protocol for follow up and treatment also in long term cancer survivors.

## Electronic supplementary material

Additional file 1: **Details about the GHT.** (DOC 34 KB)
