# Supplementary material for: Growth impairment after TBI of leukemia survivors children: a model- based investigation
Source: Theor Biol Med Model. 2014 Oct 13;11:44. doi: 10.1186/1742-4682-11-44 (PMC4213466; doi:10.1186/1742-4682-11-44)
Supplement: Supplementary file 1 — Additional file 1: Details about the GHT. (DOC 34 KB) [file 12976_2014_484_MOESM1_ESM.doc]

| **patient** | **Lenght at birth** | **Target height (cm)** | **sex** | **Somatomedins (ng/ml)** | **Other exams/associated conditions** | **Age at beginning of GH therapy** | **dose** | **Duration of therapy** |
| --- | --- | --- | --- | --- | --- | --- | --- | --- |
| 13 |  | 153.5 | F | 108 (nv 117-771) | cortisol 3,1 mcg/dl (nv 6-15); low growth velocity | 7 years and 10 months | 0,2mg/Kg/week for 7 months; 0,26 mg/Kg/ week for 6 months; 0,34 mg/Kg week for 13 months | 2 years and 2 months (interrupted for pain during walk) |
| 16 | 48 cm | 158.5 | F | 54 (nv 117-771) | low growth velocity | 6 years and 1 month | 0,1 mg/Kg/ week for 2 years and 7 months; 0,2 mg/Kg/ week from august 2006 to february 2011 | 7 years |
| 17 | n.a. | 157.5 | F | 119 (nv 117-771) |  | 8 years and 10 months | 0,18 mg/Kg/ week for 10 months; 0,27 mg/Kg/ week per 5 months ; 0,33 mg/Kg/ week for 6 months; 0,39 mg/Kg/ week from march 2003 to april 2011. | 9 years and 9 months |
| 19 | 49 cm | 169 | F | 80 (nv 117-771) | low growth velocity | 7 years and 11 months | 0,2 mg/Kg/ week from january 2008 to march 2010 | 2 years and 4 months |
| 27 | 50 cm | 166 | F | 165 (nv 235-983) |  | 12 years and 1 month | 0,17 mg/Kg/ week for 13 months; 0,22 mg/Kg/ week from January 2008 to september 2010; pause; restart from april 2011 to july 2011 | approximately 3 years and 6 months |
| 11 |  | 172 | M |  | spermatogenesis deficit | 14 years and 7 months | 0,17 mg/Kg/ week for 5 months; 0,22 mg/Kg/ week from june 2007 to january 2009 | approximately 2 years |
| 20 | 51 cm | 177.5 | M |  |  | 9 years and 11 months | 0,18 mg/Kg/ week from april 2007 to july 2011 | 4 years and 3 months |
